# Connexin/Innexin Channels in Cytoplasmic Organelles. Are There Intracellular Gap Junctions? A Hypothesis!

**DOI:** 10.3390/ijms21062163

**Published:** 2020-03-21

**Authors:** Camillo Peracchia

**Affiliations:** Department of Pharmacology and Physiology, School of Medicine and Dentistry, University Rochester, 601 Elmwood Ave., Rochester, NY 14642, USA; camillo_peracchia@urmc.rochester.edu

**Keywords:** gap junctions, connexin, innexin, calmodulin, mitochondria, cell-to-cell channels, channel gating, crayfish giant axons, liver, stomach

## Abstract

This paper proposes the hypothesis that cytoplasmic organelles directly interact with each other and with gap junctions forming intracellular junctions. This hypothesis originated over four decades ago based on the observation that vesicles lining gap junctions of crayfish giant axons contain electron-opaque particles, similar in size to junctional innexons that often appear to directly interact with junctional innexons; similar particles were seen also in the outer membrane of crayfish mitochondria. Indeed, vertebrate connexins assembled into hexameric connexons are present not only in the membranes of the Golgi apparatus but also in those of the mitochondria and endoplasmic reticulum. It seems possible, therefore, that cytoplasmic organelles may be able to exchange small molecules with each other as well as with organelles of coupled cells via gap junctions.

## 1. Introduction

In most tissues, neighboring cells directly exchange cytosolic molecules as heavy as ~1 kD via cell-to-cell channels aggregated at gap junctions [1,2,3,4]. Direct cell–cell communication (cell coupling) is a very efficient mechanism for coordinating numerous cellular activities. Indeed, abnormal cell communication is known to cause several diseases.

Cell-to-cell channels are made of the interaction of two hemichannels (connexons/innexons), each made of six proteins (connexins/innexins) that create a hydrophilic pore spanning both apposed plasma membranes and a narrow extracellular space (gap). A gating mechanism driven by nanomolar cytosolic Ca^2+^-concentrations [Ca^2+^]_i_ [3,5] via calmodulin (CaM) activation is believed to regulate channel permeability [6,7].

It is generally thought that the only function of gap junctions is to directly connect electrically and metabolically neighboring cells. It is also known that these gap junction proteins can form non-junctional hemichannels in plasma membranes. However, connexins (innexins) assembled into connexons/innexons are also present in the Golgi apparatus, mitochondria and, in some cases, the endoplasmic reticulum (ER); indeed, connexins are ubiquitously expressed in intracellular organelles [8].

The questions then are as follows: 1. Are the intracellular connexons/innexons capable of forming functional intracellular hemichannel? 2. Do intracellular connexons/innexons interact with each other to form intracellular junctions? 3. Do intracellular connexons/innexons interact with plasma-membrane gap junctions? Indeed, some intriguing findings, published over the last four-plus decades, have raised the possibility that connexin/innexin-mediated communication might also occur intracellularly between organelles, as well as between organelles and plasma membrane gap junctions via “inverted” gap junctions.

## 2. Direct Interaction between Crayfish Gap Junctions and Intracellular Vesicles

**Figure 1:**
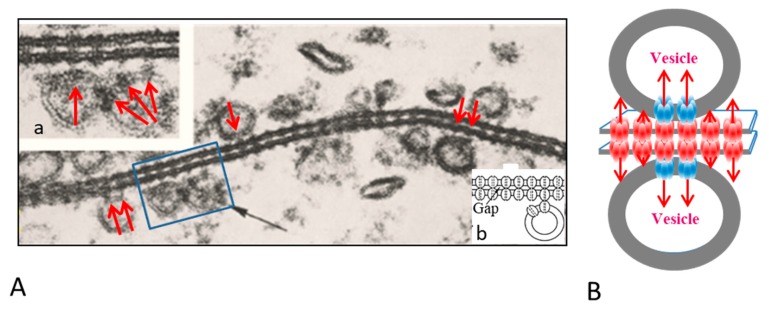
**A**. Electron micrograph showing the profile of a gap junction between crayfish lateral giant axons (**A**). The membranes display a beaded profile created by particles (innexons) that are in register, protrude from both membrane surfaces and bind to each other across the extracellular gap (**A**). The membranes are coated with 500–800 Å vesicles (**A**, black arrow) whose membrane also contains particles (**A**, red arrows), similar to junctional particles that often come in direct contact with junctional particles (**A** and insets a and b). Occasionally, neighboring vesicles appear to bind to each other via particle–particle interactions (**A**, inset a). The vesicles may directly communicate with each other and with vesicles lining on the other side of the junction (**B**). A from [9].

**Figure 2:**
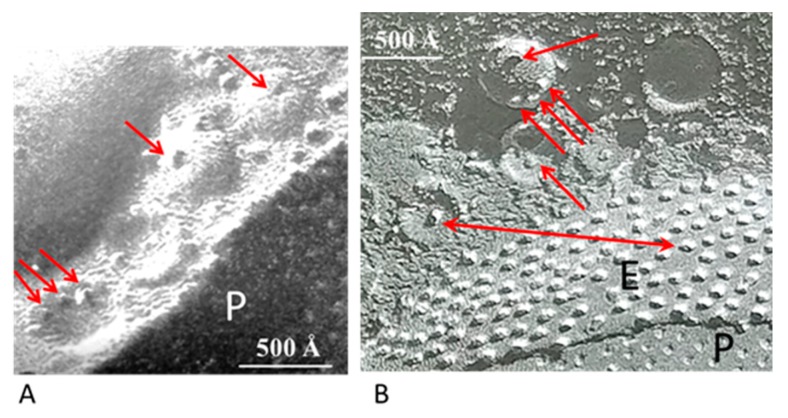
Freeze-fracture images of gap junctions between crayfish lateral giant axons (**A** and **B**). The neighboring vesicles contain particles and pits (**A** and **B**, red arrows) similar in size to the junctional particle (**B**). Often, the particles of the vesicles display a central dimple similar in size to that of the junctional particles (**B**, double-headed red arrow). P, Protoplasmic face; E, Exoplasmic face. **A** from [10].

The gap junctions of crayfish lateral giant axons are coated with 500–800 Å vesicles that line both junctional surfaces in single rows (Figure 1A) [9]. Since these junctions are electrical (electrotonic) and so, unlike chemical synapses, transmit the electrical impulse virtually without delay, these vesicles are unlikely to contain a neurotransmitter. Therefore, what could be their function? Significantly, the vesicles’ membranes often display electron-opaque particles, similar in size and spacing to junctional innexons (Figure 1A) [9] that occasionally appear to precisely interact with the cytoplasmic end of junctional innexons (Figure 1A and inset a, red arrows), forming what appear to be small intracellular junctions (Figure 1A, a and b) [9,10]. Particles similar to plasma membrane innexons are also seen in freeze-fractured vesicles (Figure 2A,B); note that particles and complementary pits (Figure 2A,B, red arrows) are similar in size to junctional particles and often display a similar central dimple (Figure 2B, double-headed red arrow) [9,10,11]. Occasionally, neighboring vesicles also appear to directly bind to each other via particle–particle interactions (Figure 1A, inset a) [9], suggesting that they may be interconnected as well.

We proposed that the vesicles contain innexons that may interact with plasma membrane innexons to establish direct communication with vesicles lining the other side of the junction (Figure 1B) [9,10,11]. If this were the case, the vesicles’ contents could be transferred to vesicles of the adjacent axon via three junctions, namely two intracellular and one intercellular. Significantly, in crayfish rectifying junctions between motor axons and either median or lateral giant axons [12,13,14], vesicles are only seen lining the gap junction of the (presynaptic) median and lateral giant axons [15,16]. This may suggest that in this case intra-vesicular molecules of the giant axon are directly transferred from presynaptic vesicles into the motor-axon’s cytosol and vice versa. Interestingly, in axons treated with chemical uncouplers, junctional membranes with tight innexon arrays, thought to contain closed channels, do not display vesicles [9,16].

## 3. Direct Interaction between Gap Junctions and Cytoplasmic Organelles

**Figure 3:**
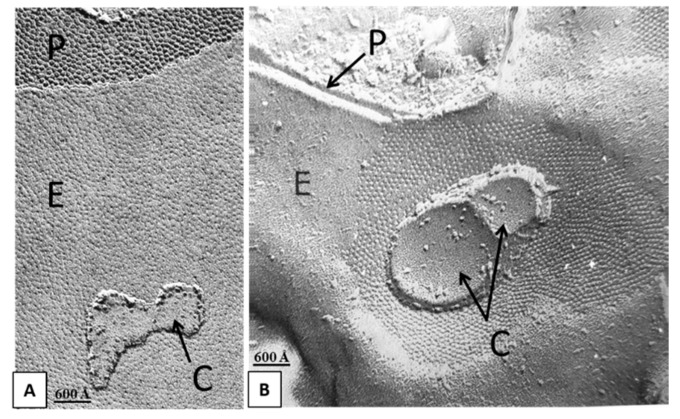
Freeze-fracture images of gap junctions between rat liver (**A**) and stomach (**B**) epithelial cells showing membranes of cytoplasmic cisterns (**C**) apparently attached to gap junction membranes. P, Protoplasmic face; E, Exoplasmic face. **A** from [18]; **B** from [17].

Over the years we have also reported images of cytoplasmic cisternal membranes tightly attached to gap junction membranes of rat liver (Figure 3A) and stomach (Figure 3B) [17,18] epithelial cells. Indeed, the close interaction between gap junctions and cytoplasmic membranes, such as smooth endoplasmic reticulum (ER) [17,19,20,21,22], rough ER (RER) [23,24] and mitochondria [23,24,25,26,27] has also been reported in various other mammalian cells. In Garant’s words [24]: “... Close association of mitochondria with the gap junctions was repeatedly observed in papillary cells and to a somewhat lesser degree in reduced ameloblasts. The most dramatic examples of this association were provided by the location of mitochondria inside the circular junctional profiles (Figures 9–11)...”. Note the bridging structures tightly connecting mitochondria and gap junction membranes in Figure 9 in [24], Figures 2, 7–9 in [25] and Figure 5c,e in [27]. In Forbes and Sperelakis’ words [25]: “... bridging structures between mitochondrial and gap junctional membranes are demonstrable in thin sections, and the appearance of replicas in which mitochondrial fragments are superimposed on gap junctions is a further indication of adhesive connection between the two structures...”.

**Figure 4:**
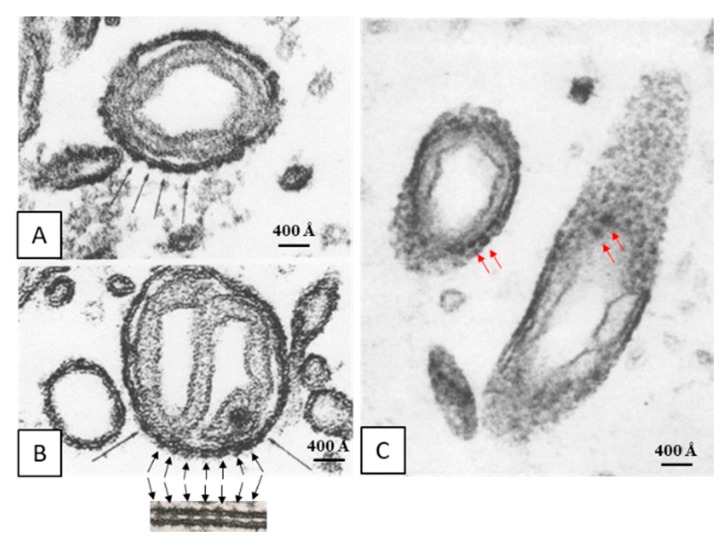
Thin sections of mitochondria in crayfish lateral giant axons. Note that the cross-sectioned outer membrane shows images of electron-opaque particles (**A** and **B**, black arrows) that are similar to gap junction particles in size and spacing (~200 Å; **B**, inset). Similar electron-opaque particles are seen in tangential sections (**C**, red arrows). From [28].

Early on, we reported the presence of electron-opaque particles in the outer membrane of crayfish mitochondria (Figure 4A–C) [28]. These particles are similar to gap junctions’ innexons in size and spacing (~200 Å; Figure 4B). In our words [28]: “... While in most of the cross sections (of mitochondrial outer membranes) the dense strata appeared very compact and did not display granularity, in tangential sections the membranes were granular because of the presence of dense particles (Figure 10). In the best preparation these particles (150 Å in size) were clearly seen separated by a distance of ~200 Å (Figures 11 and 12) and seemed to be located on the axoplasmic side of the unit membranes (Figure 11). In septal electrical synapses (lateral giant fibers) particles (innexons) of the same size were seen more clearly (Figure 13). Here, in membranes seen in face view, they were organized in a hexagonal array in which the center-to-center distance between adjacent particles was 200 Å...”.

Indeed, in recent years, connexins have been found in the inner membrane of cardiac mitochondria and in other tissues’ mitochondria [29,30,31,32,33]. A study reported that Cx43 is expressed in the outer mitochondrial membrane as well [32]. Cx32 is also present in mitochondrial membrane [34]; the mitochondrial Cx32 is thought to interact with the Cx32 of plasma membranes by an accessory protein (SFXN-1; siderioflexin-1) [34].

Gap junctions may also interact with cisterns of the Golgi apparatus because most connexins oligomerize into connexons in the Golgi apparatus. Perhaps there are also interactions with cisterns of the ER. Indeed, for some connexins this might be the case because there is evidence that Cx32 and Cx37, at least, oligomerize in the ER [35,36]. If indeed gap junctions interact with cytoplasmic organelles, perhaps organelles might interact with each other as well.

## 4. Potential Intracellular Connexin–Connexin Interactions

If indeed intracellular gap junctions exist, possible “inverted” connexin–connexin interactions might occur between opposite cytoplasmic domains such as cytoplasmic loops (CL) or COOH-termini (CT), forming CL–CL, CT–CT or CL–CT interactions. If so, this would create inverted/intracellular gap junctions (connexins linked by cytoplasmic rather that extracellular domains).

**Figure 5:**
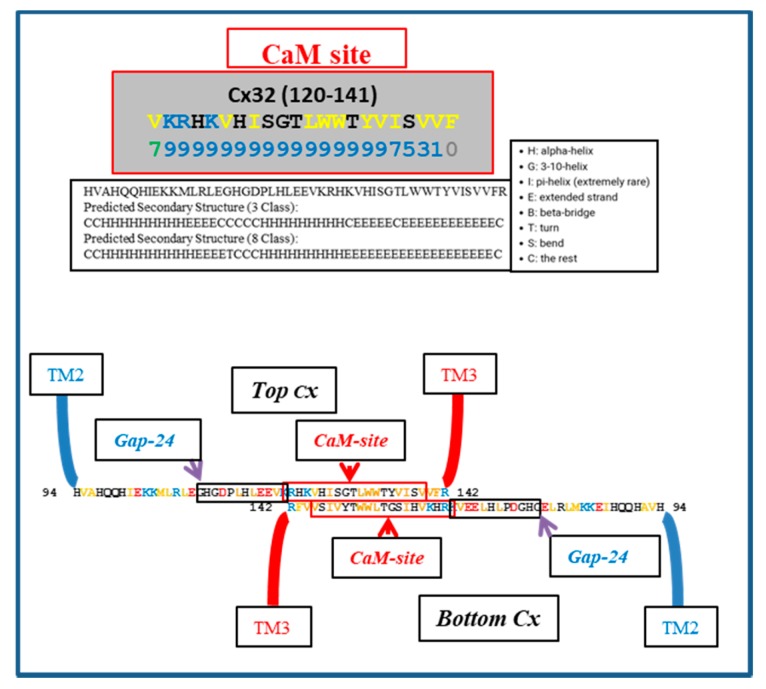
Hypothetical CL–CL interaction between Cx32 sequences. If present, the interaction might involve numerous hydrophobic residues and include the CL2′s calmodulin (CaM) binding site, which is close to the “32gap 24” amino acid chain. If this were the case, CaM would not bind to the CL2 domain. Above is the predicted secondary structure of this sequence, performed by SCRATCH Protein Predictor, School of Informatics and Computer Sciences (ICS), University of California, Irvine (UCI).

Figure 5 shows a hypothetical CL–CL interaction between Cx32 monomers; the CL–CL interaction could involve six hydrophobic residues: four valine (V) and two tryptophan (W) residues. Note that this interaction, if present, could also involve the calmodulin (CaM) binding site located in the second half of the cytoplasmic loop (CL2; Figure 5), which is close to the “32gap 24” amino acid chain [37] (Figure 5). If so, CaM would not be able to interact with its CL site, and the channels would be insensitive to Ca^2+^_i_. For a review of CaM binding sites in connexins, see [7]; the CaM-binding sites in connexins were identified by a computer program that rates the probability of CaM–Cx interactions from 0 to 9 [38].

**Figure 6:**
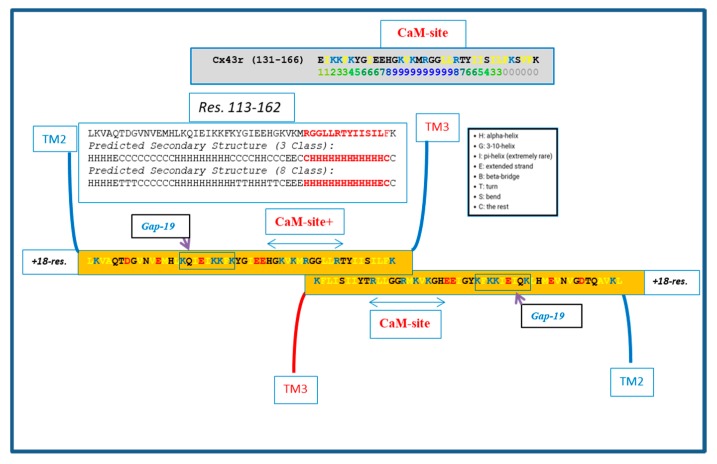
Hypothetical CL–CL interaction between Cx43 sequences. If present, the interaction might involve several hydrophobic residues and include the CL2′s calmodulin (CaM) binding site. Based on the prediction of the secondary structure (see above), the potential CL–CL interacting sequence (R148–K162) is believed to be in alpha-helical conformation. The secondary structure prediction was performed by SCRATCH Protein Predictor, School of Informatics and Computer Sciences (ICS), University of California, Irvine (UCI).

**Figure 7:**
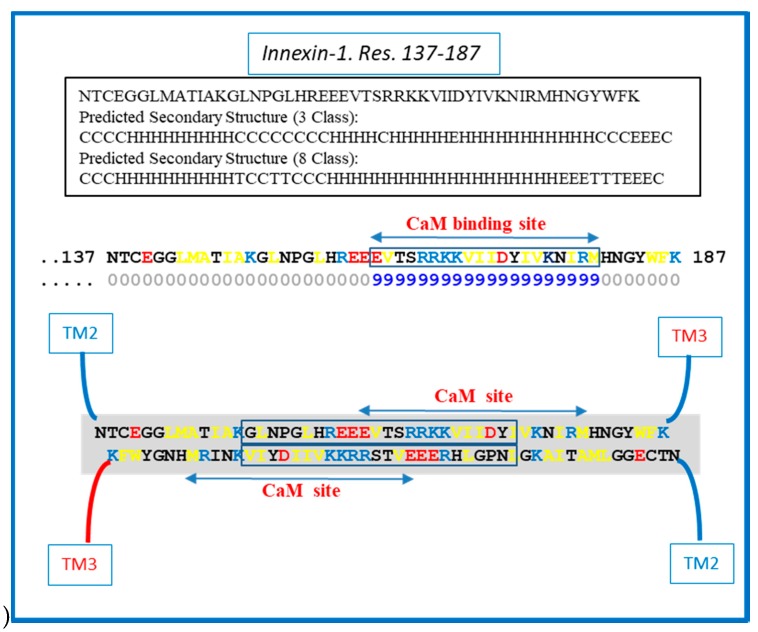
Hypothetical CL–CL interaction between innexin-1 sequences (in squares). If present, the interaction might involve hydrophobic and charged residues and include part of the CL2′s calmodulin (CaM) binding site. The prediction of the secondary structure shown above was performed by SCRATCH Protein Predictor, School of Informatics and Computer Sciences (ICS), University of California, Irvine (UCI).

Similar CL–CL interactions could also occur with Cx43 (Figure 6) innexin-1 (Figure 7) and other connexins/innexins. As for Cx32, in Cx43 and innexin-1, this potential CL–CL interaction would also interfere with CaM binding (Figure 6 and Figure 7).

We do realize that the CL–CL interactions are very hypothetical. One of the reasons why we favor the CL–CL interaction is that in this case the pores of the two connexons would be relatively well aligned. However, there is evidence that COOH-terminus (CT) tails can dimerize [39,40,41], suggesting that CT–CT interactions may be possible as well. In addition, evidence that the CL’s peptides Gap 24 [37] and Gap 19 [42,43,44] (Figure 5 and Figure 6, respectively) bind to CT suggests that CL–CT interactions could very well be involved as well.

## 5. Future Perspectives

These provocative findings should encourage one to explore in detail the largely unknown field of intracellular connexin function. One may question: Are there connexin-mediated interactions between intracellular organelles? Are there interactions between gap junctions and cytoplasmic organelles? If so, what could their function be? Would connexons/innexons be capable of interacting with other connexons/innexons by means of their cytoplasmic molecular domains? Would intracellular organelles be able to exchange molecules across gap junctions with organelles of the coupled cell? Would there be intra-mitochondrial junctions to establish communication between their matrix and the cytosol and/or other organelles? As we have proposed for crayfish vesicle/gap junction and vesicle/vesicle interactions, it might be possible that gap junction permeable molecules such as ions, amino-acids, second messengers, micro-RMAs and other nucleotides could be shared among organelles and adjacent cells via intracellular junctions. In addition, small cytosolic molecules could diffuse from cytosol to organelles and vice versa via organelles’ connexin/innexin hemichannels.

Recently, Gemel and coworkers [45] have reported that exosomes, small extracellular vesicles containing Cx43 hemichannels, are secreted by cells and could fuse with each other and/or with plasma membrane hemichannels. This would create pathways for exchange of small molecules, including cell signaling molecules, between exosomes and between exosomes and recipient cells.

A first step in testing whether intracellular junctions might form could be to study the possible molecular interaction between cytoplasmic domains. The potential interaction between gap junctions and cytoplasmic sequences could be tested by immunofluorescent microscopy and with mimetic peptides by in vitro methods, such as those used for testing the CaM binding to peptides matching potential CaM sites in connexins [37,46,47], or by other methods such as surface plasmon resonance and/or microscale thermophoresis.
